# Cox proportional hazards regression in small studies of predictive biomarkers

**DOI:** 10.1038/s41598-024-64573-9

**Published:** 2024-06-20

**Authors:** K. Jóźwiak, V. H. Nguyen, L. Sollfrank, S. C. Linn, M. Hauptmann

**Affiliations:** 1 Institute of Biostatistics and Registry Research, Brandenburg Medical School Theodor Fontane, Fehrbelliner Straße 39, 16816 Neuruppin, Germany; 2 Division of Molecular Pathology, The Netherlands Cancer Institute, Amsterdam, The Netherlands; 3 Department of Medical Oncology, The Netherlands Cancer Institute, Amsterdam, The Netherlands; 4 Department of Pathology, University Medical Center, Utrecht, The Netherlands; 5 Present Address: Leibniz Centre for Agricultural Landscape Research (ZALF), Müncheberg, Germany

**Keywords:** Predictive markers, Statistics, Tumour biomarkers

## Abstract

Predictive biomarkers are essential for personalized medicine since they select the best treatment for a specific patient. However, of all biomarkers that are evaluated, only few are eventually used in clinical practice. Many promising biomarkers may be erroneously abandoned because they are investigated in small studies using standard statistical techniques which can cause small sample bias or lack of power. The standard technique for failure time endpoints is Cox proportional hazards regression with a multiplicative interaction term between binary variables of biomarker and treatment. Properties of this model in small studies have not been evaluated so far, therefore we performed a simulation study to understand its small sample behavior. As a remedy, we applied a Firth correction to the score function of the Cox model and obtained confidence intervals (CI) using a profile likelihood (PL) approach. These methods are generally recommended for small studies of different design. Our results show that a Cox model estimates the biomarker-treatment interaction term and the treatment effect in one of the biomarker subgroups with bias, and overestimates their standard errors. Bias is however reduced and power is increased with Firth correction and PL CIs. Hence, the modified Cox model and PL CI should be used instead of a standard Cox model with Wald based CI in small studies of predictive biomarkers.

## Introduction

In past decades, much research focused on identifying characteristics of tumors and patients to optimize anticancer therapy in individual patients^1^. To improve tumor response, information like germline and tumor genetic variability, tumor (immune) environment, and lifestyle and comorbidities of patients diagnosed with cancer can be taken into account^2^. A characteristic that identifies patients who require additional systemic therapy besides local therapy (surgery, radiotherapy), i.e., indicates who needs additional therapy, is a prognostic biomarker. A characteristic that selects the most promising treatment for a specific patient, i.e., indicates how one should be treated, is a predictive biomarker^3^. Thus, predictive biomarkers are essential for personalized medicine.

Of the many evaluated biomarkers, only few reach clinical practice because of many challenges during the translational phase^4,5^. One possible concern might be the use of suboptimal statistical methods for the available biomarker data.

To identify a binary predictive biomarker, application of a statistical interaction test between the biomarker and the treatment is recommended to evaluate whether a relative benefit from a specific experimental treatment compared with a control treatment differs by biomarker level^6,7^. However, to guide a treatment choice, a qualitative rather than a quantitative interaction is needed. A qualitative interaction is present when an experimental treatment is not superior to a control treatment (i.e., is equally efficacious or worse) at one biomarker level but is superior at the other biomarker level. A quantitative interaction is present when an experimental treatment is superior to a control treatment in both biomarker levels but the magnitude of the treatment benefit differs in these subgroups^8^.

A commonly used statistical method for failure time data is the Cox proportional hazards model with a multiplicative interaction term between indicator variables of biomarker and treatment in a cohort of suitable patients^9^. Unfortunately, to obtain unbiased estimates and to detect statistically significant interactions of moderate size with sufficient statistical power, a large number of patients is required^8^, which is often not available in biomarker studies. Even if available, limited research budgets may prohibit the often costly measurement of the biomarker status in a large cohort. Consequently, too small patient series are interrogated and small sample bias as well as lack of power can lead to inconclusive results and thus perhaps to abandoning a promising biomarker.

In order to understand the small sample behavior of the Cox model^9^ for interaction analyses, we performed a simulation study in settings similar to the results of existing clinical studies on breast cancer (BC)^10–14^. We focused on properties of the biomarker-treatment interaction estimate. Additionally, we evaluated estimates of the treatment effects by biomarker level. Results of a standard Cox model were compared with a Firth-corrected Cox model, i.e., a Cox model with a modified score function^15^. Profile likelihood (PL) and Wald confidence intervals (CI) were also compared. To our knowledge, the Firth correction and PL CIs have not yet been investigated for a Cox model with an interaction term. However, they were evaluated to overcome the asymptotic bias for the estimation of prognostic effects of covariates. Heinze and Schemper^16^ and Heinze^17^ demonstrated that in small samples the Firth-corrected Cox model was superior over a standard Cox model, especially in scenarios with heavy censoring and strong covariate effects on survival. They also showed that inference should be based on PL CIs rather than on Wald-type CIs. Therefore, the aim of our study was to replicate the results of Heinze and Schemper^16^ and Heinze^17^ in settings specific to studies on predictive biomarkers. We evaluated and compared results of studies with protective, null and harmful biomarker effects and biomarkers of varying prevalence, as well as studies with varying strengths and directions of the association between the biomarker and the treatment. Our focus was to find scenarios that could indicate when data on predictive biomarker have to be analyzed with modifications of the standard Cox model.

## Methods

### Data generation

The simulation study design followed the recommendations of Morris et al.^18^ and methods described by Bender et al.^19^. *N* datasets with *n* patients each were generated. All patients were assigned to one of four combinations of biological marker *M* (low level: $M=0$; high level: $M=1$) and treatment *T* (standard treatment: $T=0$; experimental treatment: $T=1$) according to a multinomial distribution with probabilities $p_{00}$ (low marker level and standard treatment), $p_{10}$ (high marker level and standard treatment), $p_{01}$ (low marker level and experimental treatment), $p_{11}$ (high marker level and experimental treatment). The probabilities were calculated as functions of the proportion $p_T$ of patients treated with the experimental treatment, the proportion $p_M$ of patients with high marker level, and the odds ratio $\text{ OR}_{MT}$ of the association between marker and treatment^20^.

Event times $t_e$ were generated as

$$\begin{aligned} t_e&=-\frac{\textrm{log} (U_e)}{\lambda _e \textrm{exp} \left( \beta _M M+ \beta _T T+\beta _{I}MT \right) }, \end{aligned}$$

where $U_e$ was a random uniform variable on the interval [0, 1], *MT* was the product of *M* and *T*, $\text{ exp }\left( \beta _M\right) =\text{ HR}_M$ was the marker hazard ratio (HR) for high vs. low marker level among patients receiving standard treatment, $\text{ exp }\left( \beta _T\right) =\text{ HR}_T$ was the treatment HR for experimental vs. standard treatment among patients with low marker level, and $\text{ exp }\left( \beta _{I}\right) =\text{ HR}_{I}$ was the interaction HR between treatment effects in high vs. low marker level. Parameter $\lambda _e$ was a scale parameter of an exponential distribution with survival function $S(t)=\text{ exp }\left( -\lambda _e t\right)$ used to calculate baseline survival, i.e., survival of patients with low marker level receiving standard treatment. The parameter was defined as

$$\begin{aligned} \lambda _e=-\frac{1}{t_{end}}\log \left( 1-p_e\right) , \end{aligned}$$

so that the baseline proportion of events before the end of follow-up $t_{end}$ was $p_e$, i.e., $S(t_{end})=1-p_e$. Censoring time $t_c$ was generated similarly with $U_c$ as a random uniform variable on the interval [0, 1], scale parameter $\lambda _c$, the proportion $p_c$ of patients with low marker level receiving standard treatment censored before $t_{end}$ (excluding administrative censoring at the end of the study period) and $\beta _M=\beta _T=\beta _{I}=0$ to achieve non-differential censoring by marker and treatment. If $t_e \le \text{ min }(t_c, t_{end}$), the patient was specified as experiencing an event at $t_e$. Otherwise, the patient was censored at $\text{ min }(t_c, t_{end}$).

Datasets with different values for *n*, $p_M$, $p_c$, $\text{ OR}_{MT}$, $\text{ HR}_M$, $\text{ HR}_{I}$ and $N=10000$, $p_T=0.5$, $p_e=0.2$, $t_{end}=5$ years, HR$_T=1$ were generated (Table 1), and the different values, except for HR$_T$, were chosen based on real datasets presented in “Data from previous breast cancer studies”. In order to generate data with a qualitative interaction between the marker and the treatment, scenarios with equally efficacious treatments among patients with low marker levels were generated (HR$_T=1$). In addition, we evaluated scenarios with a selection of quantitative interactions as observed in real datasets, i.e., beneficial treatment effects in both marker levels but with different relative magnitude. We do not show these results but refer to them in the discussion.

**Table 1:** Parameter values used for generating data.

Parameter	Description of parameter	Value(s)
*N*	Number of simulated datasets	10,000
*n*	Number of patients per dataset	200, 300, 400, 500, 600, 800, 1000
$p_M$	Proportion of patients with high marker level	0.25, 0.5, 0.75
$p_T$	Proportion of patients treated with experimental treatment	0.5
$p_e$	Proportion of patients with low marker level receiving standard treatment who	
	experienced the event before the end of follow-up	0.2
$p_c$	Proportion of patients with low marker level receiving standard treatment censored	
	before the end of follow-up, excluding administrative censoring	0.2, 0.5
$t_{end}$	Maximum length of follow-up	5
$\text{ OR}_{MT}$	Ratio between odds of high marker level for patients treated with experimental	
	vs. standard treatment	0.5, 1, 2
$\text{ HR}_M$	Ratio of the hazard rates of event occurrence for high vs. low marker level among	
	patients receiving standard treatment, $\text{ exp }(\beta _M)$ in formula (1) and (2)	0.6, 0.8, 1, 3, 6
$\text{ HR}_T$	Ratio of the hazard rates of event occurrence for experimental vs. standard treatment	
	among patients with low marker level, $\text{ exp }(\beta _T)$ in formula (1)	1
$\text{ HR}_{I}$	Marker-treatment-interaction hazard ratio, i.e., ratio of the treatment hazard ratios	
	of event occurrence for high vs. low marker level, $\text{ exp }(\beta _I)$ in formula (1)	
	and $\text{ exp }\left( \beta _{TM_{high}}\right) /\text{exp }\left( \beta _{TM_{low}}\right)$ in formula (2)	0.25, 0.5, 0.75, 1

**Table 2:** Summary of previous breast cancer studies.

	de Boo et al.^10^	Knauer et al.^11^	Kok et al.^12^	Schouten et al.^13^	Vollebergh et al.^14^
Study design	RCT	Observational $^{\text {a}}$	RCT	Observational	RCT
Patient group	Early-stage TN	pT1-3, N0-1, M0	Premenopausal, Stage II	High-risk	Stage III, HER2−
Standard treatment	T + CEF	ET	No TAM	Conventional CT	Conventional CT
Experimental treatment	TX + CEX	ET+CT	TAM	High-dose CT	High-dose CT
Marker	BRCA1-like	MammaPrint	ER$\alpha$S118-P	BRCA1-like	BRCA1-like
Endpoint	RFS	BCSS	RFS	DFS	RFS
*n*	129	541	239	117	230
$p_M$	0.53	0.53	0.52	0.14	0.18
$p_T$	0.47	0.42	0.49	0.59	0.49
$p_e$	0.38	0.03	0.40	0.34	0.38
$p_c$	0	0.88	0	0.65	0
$\text{ OR}_{MT}$	0.92	2.34	1.48	1.70	0.79
$\text{ HR}_M$	0.67	6.60	0.86	5.39	3.51
$\text{ HR}_T$	0.23	0.56	0.83	0.87	0.68
$\text{ HR}_{I}$	1.95	0.37	0.63	0.08	0.24
$\text{ p}_{I}$	0.45	0.42	0.32	0.01	0.02

*BCSS* breast cancer-specific survival, *CT* chemotherapy, *DFS* disease-free survival, *ET* endocrine therapy, *HER2*− negative human epidermal growth factor receptor 2, *N* node stage, *M* metastasis stage, $\text{ p}_{I}$ p-value for marker-treatment interaction term, *pT* pathological tumor stage, *RCT* randomized controlled trial, *RFS* recurrence-free survival, *TAM* tamoxifen, *T + CEF* chemotherapy with docetaxel followed by cyclophosphamide-epirubicin-fluorouracil, *TN* triple-negative, *TX-CEX* chemotherapy with capecitabine-docetaxel followed by cyclophosphamide-epirubicin-fluorouracil.

$^{\text {a}}$ Pooled study of 6 observational studies.

### Data from previous breast cancer studies

We based our simulation scenarios on information from five studies of different BC subtypes, namely early-stage triple negative^10^, early^11^, premenopausal stage II^12^, high-risk^13^, and stage III negative human epidermal growth factor receptor 2^14^. The endpoints considered were either time to BC relapse or death due to any cause [recurrence-free survival (RFS), disease-free survival (DFS)] or time to death due to BC [breast cancer-specific survival (BCSS)]. The information we extracted from the datasets (Table 2) differed slightly from published results because we did not adjust analyses for prognostic or confounding variables. We also censored patients at 5 years to be able to compare values of the different parameters in our simulation scenarios across studies.

Three of the studies were randomized controlled trials and two were observational series of patients. In none of the studies was the evaluation of the predictive marker effect a primary objective. Marker measurements were obtained using archived specimens and were not available for all patients in the original study. Three studies evaluated the BRCA1-like marker, and two studies compared high-dose chemotherapy to conventional chemotherapy. The sample size varied from 117 to 541. The proportion of patients with high marker levels was about 50$\%$ in three studies, but only 14$\%$ and 18$\%$ in the other two studies. The experimental treatment was given to 42–58$\%$ of the patients, and the $\text{ OR}_{MT}$ between marker and treatment was between 0.79 and 2.34. The marker was protective among patients treated with the standard treatment in two studies ($\textrm{HR}_M=0.67$, 0.86), but harmful in the other three studies ($\text{ HR}_M=3.51$, 5.39, 6.60). In all studies, patients with the low marker level benefitted from the experimental treatment ($\textrm{HR}_T$ between 0.23 and 0.87) and in only one study was the benefit of the experimental treatment greater for patients with low vs. high levels, i.e., the marker-treatment interaction term exceeded 1 ($\text{ HR}_I=$1.95).

### Data analysis

The generated datasets were analyzed using a standard Cox proportional hazards model^9^ with hazard function

1 $$\begin{aligned} h(t;T,M)=h_0(t) \text{ exp }\left( \beta _{M} M+ \beta _{T} T+\beta _{I}MT\right) , \end{aligned}$$

where $h_0$ was the baseline hazard function. Note that, if $\beta _{I}=0$, the joint effect of marker and treatment is multiplicative, i.e., the HR of a patient with a high marker level and experimental treatment vs. a patient with a low marker level and standard treatment is the product of $\text{ exp }\left( \beta _M\right)$ and $\text{ exp }\left( \beta _T\right)$. This situation is often referred to as absence of interaction. If $\beta _{I} \ne 0$, the joint effect is super- ($\beta _{I}>0$) or submultiplicative ($\beta _{I}<0$), and this is usually referred to as interaction.

Additionally, we used the following parametrization of model (1)

2 $$\begin{aligned} h(t;T,M)=h_0(t) \text{ exp }\left( {\beta _M M+ \beta _{TM_{low}} TM_{low}+\beta _{TM_{high}} TM_{high}}\right) \end{aligned}$$

for the evaluation of the treatment effect by marker level. $TM_{low}$ and $TM_{high}$ were binary variables indicating patients receiving experimental treatment in the two marker levels, i.e., $TM_{low}=1$ if $M=0$ and $T=1$, and $TM_{low}=0$ otherwise; $TM_{high}=1$ if $M=1$ and $T=1$, and $TM_{high}=0$ otherwise. Here, $\text{ exp }\left( \beta _{TM_{low}}\right) =\text{ HR}_{TM_{low}}$ and $\text{ exp }\left( \beta _{TM_{high}}\right) =\text{ HR}_{TM_{high}}$ were the HRs for experimental vs. standard treatment in subgroups of low and high marker levels, respectively. All datasets were also analyzed by a bias-eliminating approach originally developed by Firth^15^ for generalized linear models and later implemented in Cox regression^16^. In contrast to the standard Cox model, a Firth-corrected Cox model provides finite HR estimates for monotone likelihoods, i.e., when the likelihood function does not have a unique maximum and the parameter estimate of a Cox model diverges with infinite standard error. For example, in our model with the interaction term, the problem can occur when there is no patient with an event in at least one of the four marker-treatment combination subgroups. Additionally, with monotone likelihoods, maximum likelihood estimate is infinite and the likelihood function becomes highly asymmetric leading to unsuitable CI obtained based on the Wald method which assumes a normal distribution of the maximum likelihood estimate. As an alternative, the PL method for CI construction is based on the asymptotic $\chi ^2$ distribution of the log likelihood ratio test statistic^21^. Therefore, we calculated and compared results obtained with 95% CI according to Wald and PL methods.

All scenarios were summarized by calculating (i) bias $\frac{1}{N_c}\sum _{j=1}^{N_c}\hat{\beta }_{I,j}-\beta _{I}$ or relative bias $\frac{1}{N_c}\sum _{j=1}^{N_c}\frac{\hat{\beta }_{I,j}-\beta _{I}}{|\beta _{I}|}$, (ii) relative % error in model standard error (ModSE) $100\left( \frac{\widehat{\text{ ModSE }}}{\widehat{\text{ EmpSE }}}-1\right)$, which was the ratio between ModSE $\sqrt{\frac{1}{N_c}\sum _{j=1}^{N_c} \widehat{\text{ Var }}\left( \hat{\beta }_{I,j}\right) }$ and empirical standard error (EmpSE) $\sqrt{\frac{1}{N_c-1}\sum _{j=1}^{N_c}\left( \hat{\beta }_{I,j}-\bar{\beta }_{I}\right) ^2}$, (iii) coverage of the CI $\frac{1}{N_c}\sum _{j=1}^{N_c}{} \mathbf{{1}}\left( \hat{\beta }_{l,j}\le \beta _{I}\le \hat{\beta }_{u,j}\right)$, where $\hat{\beta }_{l,j}$ was the lower bound and $\hat{\beta }_{u,j}$ was the upper bound of the 95% CI around $\hat{\beta }_{I,j}$, and (iv) type I error or power $\frac{1}{N_c}\sum _{j=1}^{N_c}{} \mathbf{{1}}(p_j\le \alpha )$, where $p_j$ was the p-value for the test $\beta _{I}=0$ obtained with the j-th dataset and $\alpha =0.05$ was the significance level. In all formulas, $N_c$ was the number of converged models, $\beta _{I}$ was the true value of the coefficient of the interaction term, $\hat{\beta }_{I,j}$ was the estimate of the interaction coefficient in the j-th dataset, $\bar{\beta }_{I}$ was the mean of all $\hat{\beta }_{I,j}$ and $\textbf{1}$ was an indicator function. Standard errors were based on the Hessian matrix. Coverage, type I error and power were calculated using both Wald and PL methods since these measures depend on the method of CI calculation. The bias and relative percentage error in standard error were additionally calculated for the treatment effect in the subgroups of low and high marker levels separately. All performance measures were defined as in^18^.

Simulation scripts were written in R version 4.3.1 using the coxphf function of the coxphf package version 1.13.4^22^ and are available on request from the first author. The maximum number of iterations (maxiter) was set to 1000 and the maximum step size (maxstep) was 0.01. If the actual number of iterations for a model fit was less than the prespecified maximum number of iterations, a model was considered converged and the estimation of the interaction term, treatment effects and their standard errors were used for summary statistics. If convergence was not reached, the dataset was discarded from summary statistics. Moreover, if a generated dataset had no events in more than one combination of marker and treatment, the dataset was not used in summary statistics irrespective of convergence. PL-based power and coverage were calculated from datasets with overall model convergence and with convergence of the confidence bound required to determine whether or not the PL CI included zero or the true parameter value. However, in additional analyses, coverage and power for both CI methods were calculated including results from non-converged models as not rejecting the null hypothesis and covering the true value.

## Simulation results

### Cox model

**Figure 1:**
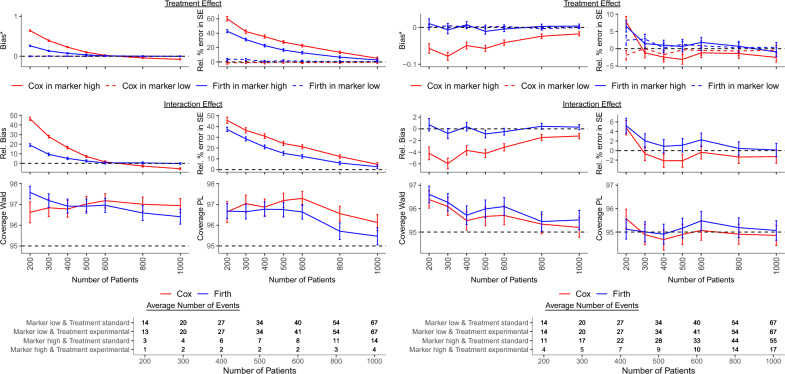
Results of the simulation study for a protective ($\text{ HR}_M=0.6$, left panel) and a harmful ($\text{ HR}_M=3$, right panel) marker effect among patients treated with the standard treatment. The treatment HRs were $\text{ HR}_{TM_{low}}=1$ and $\text{ HR}_{TM_{high}}=0.25$, the interaction HR was $\text{ HR}_{I}=0.25$, the OR between marker and treatment was $\text{ OR}_{MT}=1$, the proportion of patients with high marker level was $p_{M}=0.25$, and the proportion of censored patients with low marker level receiving standard treatment was $p_c=0.2$. $^*$Curves of bias in marker low group for Cox and Firth model overlap. *HR* hazard ratio, *OR* odds ratio, *PL* profile likelihood, *Rel.* relative, *SE* standard error.

**Figure 2:**
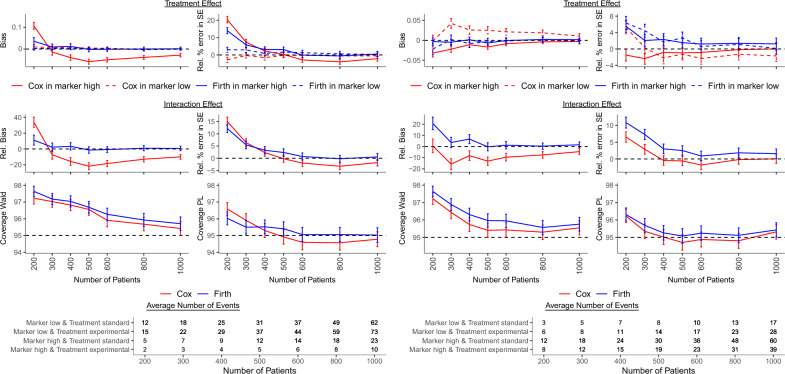
Results of the simulation study for 25% ($p_M=0.25$, left panel) and 75% ($p_M=0.75$, right panel) of patients with high marker level. The treatment HRs were $\text{ HR}_{TM_{low}}=1$ and $\text{ HR}_{TM_{high}}=0.75$, the interaction HR was $\text{ HR}_{I}=0.75$, the marker effect among patients treated with the standard treatment was $\text{ HR}_M=0.8$, the OR between marker and treatment was $\text{ OR}_{MT}=0.5$, and the proportion of censored patients with low marker level receiving standard treatment was $p_c=0.2$. *HR* hazard ratio, *OR* odds ratio, *PL* profile likelihood, *Rel.* relative, *SE* standard error.

**Figure 3:**
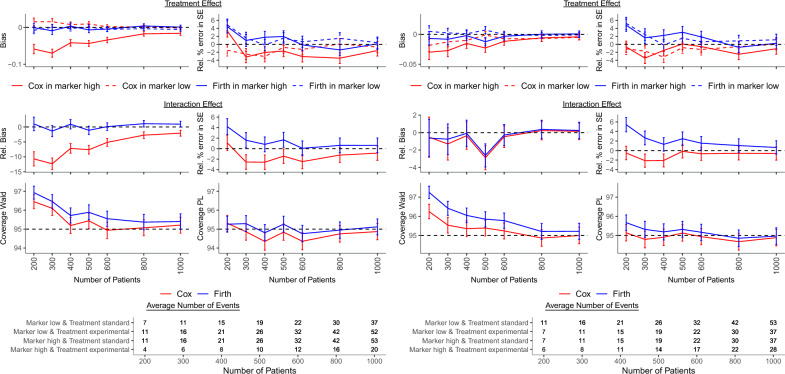
Results of the simulation study when fewer ($\text{ OR}_{MT}=0.5$, left panel) and more ($\text{ OR}_{MT}=2$, right panel) patients with high marker level received experimental in comparison to standard treatment and the proportion of patients with high marker level was $p_M=0.5$. The treatment HRs were $\text{ HR}_{TM_{low}}=1$ and $\text{ HR}_{TM_{high}}=0.5$, the interaction HR was $\text{ HR}_{I}=0.5$, the marker effect among patients treated with the standard treatment was $\text{ HR}_M=1$, and the proportion of censored patients with low marker level receiving standard treatment was $p_c=0.2$. *HR* hazard ratio, *PL* profile likelihood, *Rel.* relative, *SE* standard error.

Relative bias of the interaction effect estimate was towards and away from the null, and its magnitude depended mostly on the number of patients per dataset and the number of events in the different marker-treatment combinations, the marker effect among patients treated with the standard treatment and the proportion of patients with high marker level. Bias was usually between − 10% and 10% and monotonically approached zero for sample sizes larger than 600 (Figs. 1, 2, 3, Tables 3, 4, 5), while for selected scenarios and smaller numbers of patients, it was high and ranged up to 72% when the proportion of patients with low marker level who received standard treatment and were censored before the end of follow-up, $p_c$, was 20$\%$ (Supplementary Table S1). Bias was also within [− 10%, 10%] when the marker had a harmful effect on survival among patients treated with the standard treatment, i.e., $\text{ HR}_M>1$, but more severe bias occurred when the marker had a protective or no effect on survival in this subgroup of patients, i.e., $\text{ HR}_M\le 1$, and the smaller the $\text{ HR}_M$ the larger the bias (Fig. 1, Table 3). What is more, higher bias was often observed for lower proportions of patients with high marker level (Fig. 2, Table 4). Nonetheless, in scenarios with $\text{ HR}_{I}=1$, i.e., with no interaction effect, bias of the interaction coefficient was always within the interval [− 0.1, 0.1] for all parameters and sample sizes (Supplementary Table S2). Thus, when the marker had a harmful effect on survival among patients treated with the standard treatment, or a high proportion of patients had a high marker level, or there was no interaction effect, bias of the interaction effect estimate was usually acceptable.

**Table 3:** Results of the simulation study for a protective ($\text{ HR}_M=0.6$), a null ($\text{ HR}_M=1$) and a harmful ($\text{ HR}_M=3$) marker effect among patients treated with the standard treatment. The treatment HRs were $\text{ HR}_{TM_{low}}=1$ and $\text{ HR}_{TM_{high}}=0.25$, the interaction HR was $\text{ HR}_{I}=0.25$, the OR between marker and treatment was $\text{ OR}_{MT}=1$, the proportion of patients with high marker level was $p_{M}=0.25$, and the proportion of censored patients with low marker level receiving standard treatment was $p_c=0.2$.

		Bias$^\text {a}$						Coverage (%)	Power (%)	
	n	$\widehat{\beta }_{TM_{low}}$	SE($\widehat{\beta }_{TM_{low}}$)	$\widehat{\beta }_{TM_{high}}$	SE($\widehat{\beta }_{TM_{high}}$)	$\widehat{\beta }_{I}$	SE($\widehat{\beta }_{I}$)	Wald (PL)	Wald (PL)	N$_{c}$
HR$_M=0.6$										
Cox	200	0	$-$ 1.2	0.6	60.2	46.5	45.7	96.6 (96.6)	2.5 (5.0)	4912
	400	0	$-$ 1.2	0.2	35.3	16.4	31.0	96.8 (96.9)	12.6 (23.1)	7671
	600	0	$-$ 0.7	0	22.8	1.6	21.2	97.2 (97.3)	33.0 (46.0)	8895
Firth	200	0	3.6	0.3	42.8	19.0	37.3	97.6 (96.7)	2.4 (11.3)	9703
	400	0	0.8	0.1	22.8	5.2	21.1	96.9 (96.8)	12.2 (29.3)	9995
	600	0	1.2	0	12.7	0.5	12.3	97.0 (96.6)	31.6 (46.6)	10,000
HR$_M=1$										
Cox	200	0	$-$ 1.1	0.3	39.3	23.9	30.0	96.6 (96.7)	8.5 (15.2)	6950
	400	0	$-$ 1.4	0	19.4	0	16.8	97.2 (97.1)	35.6 (46.3)	9129
	600	0	$-$ 0.6	$-$ 0.1	6.9	$-$ 5.7	6.5	96.8 (96.0)	61.6 (68.1)	9750
Firth	200	0	3.2	0.1	28.3	8.6	24.8	97.2 (96.6)	7.5 (21.5)	9969
	400	0	0.6	0	11.0	0.8	9.9	97.0 (96.1)	33.7 (46.5)	9999
	600	0	1.1	0	4.3	$-$ 0.5	4.2	96.5 (95.3)	59.3 (65.8)	10,000
HR$_M=3$										
Cox	200	0	$-$ 1.9	$-$ 0.1	7.8	$-$ 4.2	4.8	96.4 (95.6)	44.7 (50.2)	9724
	400	0	$-$ 1.7	0	$-$ 2.4	$-$ 3.7	$-$ 2.1	95.5 (94.7)	79.4 (81.0)	9991
	600	0	$-$ 0.5	0	$-$ 1.2	$-$ 3.2	$-$ 0.4	95.7 (95.1)	93.8 (94.3)	10,000
Firth	200	0	2.5	0	6.5	0.7	5.2	96.6 (95.1)	41.5 (48.8)	10,000
	400	0	0.2	0	1.0	0.4	0.9	95.7 (94.9)	77.5 (80.0)	10,000
	600	0	0.8	0	1.8	$-$ 0.5	2.2	96.1 (95.5)	93.3 (94.0)	10,000

$^\text {a}$Bias for $\widehat{\beta }_{TM_{low}}$, $\widehat{\beta }_{TM_{high}}$ and relative bias (%) for SE($\widehat{\beta }_{TM_{low}}$), SE($\widehat{\beta }_{TM_{high}}$), $\widehat{\beta }_{I}$, SE($\widehat{\beta }_{I}$).

Other parameters: $$\text{ HR}_{TM_{low}}=1$$, $$\text{ HR}_{TM_{high}}=0.25$$, $$\text{ HR}_{I}=0.25$$, $$\text{ OR}_{MT}=1$$, $$p_{M}=0.25$$, $$p_c=0.2$$.

*HR* hazard ratio, *n* number of patients per dataset, $N_c$ number of converged models, *OR* odds ratio, *PL* profile likelihood, *SE* standard error.

**Table 4:** Results of the simulation study for 25% ($p_M=0.25$), 50% ($p_M=0.5$) and 75% ($p_M=0.75$) of patients with high marker level. The treatment HRs were $\text{ HR}_{TM_{low}}=1$ and $\text{ HR}_{TM_{high}}=0.75$, the interaction HR was $\text{ HR}_{I}=0.75$, the marker effect among patients treated with the standard treatment was $\text{ HR}_M=0.8$, the OR between marker and treatment was $\text{ OR}_{MT}=0.5$, and the proportion of censored patients with low marker level receiving standard treatment was $p_c=0.2$.

		Bias$^\text {a}$						Coverage (%)	Power (%)	
	n	$\widehat{\beta }_{TM_{low}}$	SE($\widehat{\beta }_{TM_{low}}$)	$\widehat{\beta }_{TM_{high}}$	SE($\widehat{\beta }_{TM_{high}}$)	$\widehat{\beta }_{I}$	SE($\widehat{\beta }_{I}$)	Wald (PL)	Wald (PL)	N$_{c}$
p$_{M}=0.25$										
Cox	200	0	$-$ 2.7	0.1	20.4	33.8	15.0	97.2 (96.6)	1.8 (3.2)	8692
	400	0	$-$ 1.5	0	2.3	$-$ 15.7	2.3	96.8 (95.3)	3.8 (6.6)	9855
	600	0	$-$ 0.2	0	$-$ 2.8	$-$ 18.4	$-$ 2.0	95.9 (94.6)	6.2 (8.8)	9978
Firth	200	0	3.0	0	14.0	11.2	12.2	97.6 (96.0)	1.4 (4.2)	9946
	400	0	0.9	0	3.1	3.4	3.3	97.0 (95.5)	3.1 (6.0)	9999
	600	0	1.3	0	$-$ 0.1	$-$ 0.8	0.7	96.3 (95.1)	5.0 (7.4)	10000
p$_{M}=0.5$										
Cox	200	0	$-$ 1.4	0	$-$ 1.4	$-$ 20.3	$-$ 1.7	95.9 (94.7)	4.9 (7.1)	9900
	400	0	$-$ 3.0	0	$-$ 2.6	$-$ 11.9	$-$ 3.1	95.0 (94.5)	7.9 (9.2)	9999
	600	0	$-$ 1.3	0	$-$ 2.4	$-$ 8.2	$-$ 2.0	94.9 (94.5)	10.2 (11.1)	10,000
Firth	200	0	4.9	0	2.7	2.9	3.6	96.8 (95.1)	3.5 (6.0)	9998
	400	0	$-$ 0.1	0	1.4	1.7	0.5	95.7 (94.7)	6.3 (7.9)	10,000
	600	0	0.7	0	0.2	0.5	0.3	95.3 (94.9)	8.7 (10.1)	10,000
p$_{M}=0.75$										
Cox	200	0	5.2	0	$-$ 1.5	0.6	6.6	97.2 (96.2)	2.7 (4.6)	9477
	400	0	$-$ 2.2	0	$-$ 0.8	$-$ 8.2	$-$ 0.5	95.7 (95.0)	5.9 (7.6)	9911
	600	0	$-$ 2.3	0	$-$ 0.8	$-$ 9.6	$-$ 1.8	95.4 (94.9)	8.8 (10.1)	9984
Firth	200	0	6.3	0	5.5	20.7	10.9	97.6 (96.3)	2.1 (4.0)	9792
	400	0	1.7	0	2.3	6.7	3.0	96.3 (95.3)	4.9 (6.6)	9947
	600	0	0.6	0	1.2	1.1	0.9	95.9 (95.2)	7.5 (9.1)	9988

$^\text {a}$Bias for $\widehat{\beta }_{TM_{low}}$, $\widehat{\beta }_{TM_{high}}$ and relative bias (%) for SE($\widehat{\beta }_{TM_{low}}$), SE($\widehat{\beta }_{TM_{high}}$), $\widehat{\beta }_{I}$, SE($\widehat{\beta }_{I}$).

Other parameters: $$\text{ HR}_{TM_{low}}=1$$, $$\text{ HR}_{TM_{high}}=0.75$$, $$\text{ HR}_{I}=0.75$$, $$\text{ HR}_M=0.8$$, $$\text{ OR}_{MT}=0.5$$, $$p_c=0.2$$.

*HR* hazard ratio, *n* number of patients per dataset, $N_c$ number of converged models, *OR* odds ratio, *PL* profile likelihood, *SE* standard error.

Relative percentage error of the estimated standard error of the interaction coefficient was predominantly positive and in general its magnitude behaved similarly as the relative bias of the interaction coefficient. Scenarios with high relative bias of the interaction coefficient generally showed also high bias of its standard error (Figs. 1, 2, 3, Tables 3, 4, 5).

The most extreme bias of the estimated interaction effect (up to 72% with $p_c=0.2$) and its standard error (up to 48% with $p_c=0.2$) was observed in scenarios with a protective marker of low prevalence, strong negative interaction, and a higher or lower prevalence of marker-positive patients in the standard vs. experimental treatment group. Both biases were positive resulting in values of the interaction effect being biased towards the null and overestimated standard error, i.e., leading to smaller or no differences in the benefit from an experimental treatment compared with a control treatment by marker level and wider CIs for the comparison of the benefit. For such a combination of parameters the incidence of the event was very low in the subgroup of patients with a high marker level and experimental treatment. Subsequently, many small datasets generated under such scenarios did not have events in this subgroup so that models failed to converge (Supplementary Table S1). In general, the smaller the values of $\text{ HR}_M$ and $p_M$, and $\text{ OR}_{MT}$ being away from 1, the larger was the number of non-converged models which ranged up to 59% (data not shown), particularly for small sample sizes.

A small number of events in at least one marker-treatment combination and positive bias of the standard error of the interaction effect estimate led to high and overestimated standard error. This resulted in very wide confidence intervals and consequently overcoverage of Wald-type 95% CIs, while coverage obtained with the PL method approached the nominal level even for smaller sample sizes (Figs. 1, 2, 3, Tables 3, 4, 5, Supplementary Table S1). Under the null, overcoverage of CIs co-occurred with a type I error below the nominal 5% level, while for scenarios with coverage close to 95%, the type I error was also close to 5% (Supplementary Table S2).

As expected, power increased with increasing sample size, higher values of the $\text{ HR}_M$ and stronger interaction effect. For a given sample size, it was usually highest for markers with 50% prevalence and lowest for markers with low prevalence (25%) when $\text{ HR}_M \le 1$ and for markers with high prevalence (75%) when $\text{ HR}_M>1$. Power was relatively independent of the association between marker and treatment, and usually larger when based on the PL vs. Wald CI. In general, a large number of patients, a strong interaction effect or a strongly harmful marker (large $\text{ HR}_M$) were needed to reach 80% power (Tables 3, 4, 5, Supplementary Table S1).

The treatment effect for the low marker level and its standard error were estimated without bias in all scenarios. However, the treatment effect and its standard error for the high marker level were estimated without or with minor bias only in scenarios with negligible bias of the interaction effect and its standard error. In other scenarios, the bias of the interaction effect and its standard error caused corresponding and similarly behaving bias of the estimates of the treatment effect and its standard error for the high marker level. Since the bias was positive or negative, both false nagative and false positive results were possible (Figs. 1, 2, 3, Tables 3, 4, 5, Supplementary Tables S1–S2). Note that we presented bias of the treatment effect for the high marker level, but its relative bias was very close to the relative bias of the interaction effect because the treatment effect for the low marker level was unbiased.

**Table 5:** Results of the simulation study when fewer ($\text{ OR}_{MT}=0.5$), the same ($\text{ OR}_{MT}=1$) and more ($\text{ OR}_{MT}=2$) patients with high marker level received experimental in comparison to standard treatment and the proportion of patients with high marker level was $p_M=0.5$. The treatment HRs were $\text{ HR}_{TM_{low}}=1$ and $\text{ HR}_{TM_{high}}=0.5$, the interaction HR was $\text{ HR}_{I}=0.5$, the marker effect among patients treated with the standard treatment was $\text{ HR}_M=1$, and the proportion of censored patients with low marker level receiving standard treatment was $p_c=0.2$.

		Bias$^\text {a}$						Coverage (%)	Power (%)	
	n	$\widehat{\beta }_{TM_{low}}$	SE($\widehat{\beta }_{TM_{low}}$)	$\widehat{\beta }_{TM_{high}}$	SE($\widehat{\beta }_{TM_{high}}$)	$\widehat{\beta }_{I}$	SE($\widehat{\beta }_{I}$)	Wald (PL)	Wald (PL)	N$_{c}$
OR$_{MT}=0.5$										
Cox	200	0	$-$ 1.5	$-$ 0.1	3.3	$-$ 10.7	1.1	96.4 (95.3)	10.9 (15.2)	9792
	400	0	$-$ 3.0	0	$-$ 2.0	$-$ 7.2	$-$ 2.6	95.2 (94.3)	23.8 (26.7)	9994
	600	0	$-$ 1.3	0	$-$ 3.1	$-$ 5.2	$-$ 2.5	94.9 (94.4)	34.6 (36.9)	10,000
Firth	200	0	4.8	0	4.4	1.0	4.2	96.9 (95.3)	8.1 (13.4)	10,000
	400	0	$-$ 0.2	0	1.8	0.9	0.8	95.7 (94.8)	20.3 (24.1)	10,000
	600	0	0.6	0	$-$ 0.1	0.1	0.1	95.6 (94.8)	31.6 (34.5)	10,000
OR$_{MT}=1$										
Cox	200	0	$-$ 1.4	$-$ 0.1	$-$ 0.1	$-$ 7.4	$-$ 0.4	96.6 (95.1)	13.0 (16.0)	9907
	400	0	$-$ 2.8	0	$-$ 1.7	$-$ 4.2	$-$ 2.5	95.4 (94.6)	25.9 (27.4)	9999
	600	0	$-$ 0.9	0	$-$ 0.9	$-$ 2.7	$-$ 1.0	95.1 (94.7)	36.9 (37.9)	10,000
Firth	200	0	5.0	0	4.0	$-$ 1.0	4.5	97.3 (95.5)	10.6 (14.8)	9998
	400	0	0	0	2.3	$-$ 0.1	1.0	96.0 (95.2)	24.0 (26.3)	9999
	600	0	1.0	0	1.7	$-$ 0.1	1.2	95.6 (95.1)	35.2 (37.0)	10,000
OR$_{MT}=2$										
Cox	200	0	$-$ 1.1	0	$-$ 0.7	$-$ 0.5	$-$ 0.5	96.3 (95.1)	13.2 (14.6)	9901
	400	0	$-$ 3.2	0	$-$ 1.5	$-$ 0.4	$-$ 2.1	95.4 (94.9)	26.2 (26.5)	9979
	600	0	$-$ 1.3	0	$-$ 0.6	$-$ 0.5	$-$ 0.7	95.2 (94.9)	36.6 (36.7)	9990
Firth	200	0	5.2	0	4.8	$-$ 0.6	5.4	97.2 (95.7)	11.8 (14.7)	9954
	400	0	$-$ 0.2	0	2.2	$-$ 0.1	1.3	96.1 (95.2)	25.6 (27.0)	9980
	600	0	0.6	0	1.8	$-$ 0.3	1.5	95.8 (95.2)	36.2 (37.3)	9990

$^\text {a}$Bias for $\widehat{\beta }_{TM_{low}}$, $\widehat{\beta }_{TM_{high}}$ and relative bias (%) for SE($\widehat{\beta }_{TM_{low}}$), SE($\widehat{\beta }_{TM_{high}}$), $\widehat{\beta }_{I}$, SE($\widehat{\beta }_{I}$)

Other parameters: $$\text{ HR}_{TM_{low}}=1$$, $$\text{ HR}_{TM_{high}}=0.5$$, $$\text{ HR}_{I}=0.5$$, $$\text{ HR}_M=1$$, $$p_M=0.5$$, $$p_c=0.2$$

*HR* hazard ratio, *n* number of patients per dataset, $N_c$ number of converged models, *PL* profile likelihood, *SE* standard error.

Increasing the proportion of patients with low marker level who received standard treatment and were censored before the end of follow-up, from 20 to 50$\%$, resulted in a slightly larger bias in all estimates, a lower power but also a larger number of non-converged models. It did not change, however, the general performance of the model (Fig. 1, Table 3, Supplementary Fig. S1, Supplementary Table S3). The number of patients per dataset, the marker effect among patients treated with the standard treatment and the proportion of patients with high marker level seemed to have a bigger impact on the performance measures of converging models. However, a large number of models fail to converge.

### Cox model with Firth correction

Virtually unbiased interaction estimates were obtained when (i) the marker was harmful among patients receiving standard treatment, (ii) more patients with high marker levels received experimental vs. standard treatment, (iii) no interaction was present between the marker and the treatment, or (iv) sample size was larger than 400. Bias of the interaction effect estimate occurred when the marker was protective among patients with the standard treatment, marker prevalence was low, the proportion of patients with high marker level was equal or higher with standard vs. experimental treatment, and the sample size did not exceed 400. In these situations, bias was also observed for the standard error (Figs. 1, 2, 3, Tables 3, 4, 5, Supplementary Tables S1–S2).

Convergence of models was very high for all scenarios and coverage of PL CIs was mostly at nominal level. Overcoverage occurred when the interaction effect or the standard error estimate was biased. Coverage of Wald CIs was generally higher than nominal. Under the null, the scenarios with overcoverage suffered from subnominal type I error rates (Figs. 1-3, Tables 3, 4, 5, Supplementary Tables S1–S2).

In all scenarios, the treatment effect and its standard error among patients with low marker levels was unbiased. Estimation of the treatment effect and its standard error in patients with high marker levels, which generally had fewer patients and a lower incidence than the low marker level, was biased when the interaction term and its standard error were biased (Figs. 1, 2, 3, Tables 3, 4, 5, Supplementary Tables S1–S2).

### Comparison of standard and Firth corrected Cox model

When comparing Cox and Firth corrected Cox model, bias was absent for sample sizes exceeding 400 with the Firth correction vs. the standard Cox model which resulted in biased estimates for sample sizes up to 600. Moreover, coverage of PL CIs was more often at nominal level and power was usually larger when a Firth correction with PL CI was applied. Additionally, the modified Cox model converged more often resulting in estimation of treatment and interaction effects in scenarios which could not be analyzed with a standard Cox model due to an insufficient number of observed events. For particular scenarios, the number of converged models was particularly higher for the modified Cox model. The higher the censoring rate the higher the difference in the number of converged models between the two approaches (Figs. 1, 2,3, Tables 3, 4, 5, Supplementary Fig. S1, Supplementary Tables S1–S3). Thus, generally implementing the Firth correction improved estimation substantially. In just a few scenarios with a protective and highly prevalent marker, a weak interaction effect and a small sample size, the Firth correction did not improve estimation (Fig. 2, Table 4).

Differences between the Firth corrected and standard Cox model became even more apparent when performance measures were obtained including results from non-converged models. Power decreased and coverage increased for the standard approach and the larger the number of non-convergence the larger the change in the performance measures. The two measures, however, were rather stable for the Firth corrected Cox model (data not shown).

Firth corrected Cox models converged usually in more than 95% of the datasets, while for the standard Cox model this was substantially less and under 50% for some scenarios. The Firth corrected Cox model performed well when results from all converging models were evaluated. Limited to results from those data sets for which both methods converged, relative bias of the (negative) interaction term in a Firth corrected Cox model was larger than for the standard Cox model in scenarios where its bias was positive. In this case, the estimate of the standard Cox model was biased towards the null and the Firth corrected estimate was even more attenuated. The standard error was then larger and the power was smaller with the Firth correction. On the other hand, in scenarios where the standard Cox model estimated the interaction coefficient with a negative bias, i.e., an overestimate, the Firth corrected estimate was less overestimated (data not shown).

## Discussion

Although the evaluation of treatment heterogeneity is a field of active research and many different approaches have been proposed^23–26^, the analysis of most clinical studies with failure time endpoints relies on informal marker-specific comparisons of survival curves by treatment or formal Cox regression with a multiplicative interaction term between marker and treatment^27^. The aim of our study was to understand the properties of the latter commonly used approach in the specific situation of BC and to identify easy-to-use modifications to improve its performance.

We showed that Cox regression yields biased results for sample sizes under 600 patients in particular settings specific to studies on predictive biomarkers, and generally overestimates the standard error of the interaction coefficient. Bias is particularly severe if few events occur in one of the four marker-treatment combinations, e.g., if the marker group with the greatest treatment benefit is small because the marker is rare, if the marker is protective and additionally decreases the event rate, or if the interaction is strong and leads to a greater treatment benefit and therefore smaller event rate. We also showed that simple modifications of the analytic method, namely a Cox model whose score function is modified with a Firth correction and CIs obtained with a PL approach, reduce bias of the interaction coefficient and marker-specific treatment effects substantially, lead to nominal coverage of CIs and increase power. Moreover, the modified Cox model converged usually in more than 95% of the datasets and much more often than the standard Cox model which converged in less than 50% of the time for some scenarios. That means that the modifications allow estimation of treatment and interaction effects in situations where the commonly used statistical model does not provide any results. When the standard Cox model converged, Firth corrected results were more or less biased than standard Cox, depending on the direction of bias. Since the direction of bias is unknown for real datasets, the results we obtained suggest that the Firth correction and PL CIs should be used instead of a standard Cox model with Wald based CI for the analysis of predictive markers. The modifications are implemented in standard statistical software packages, for example, in SAS in the PROC PHREG procedure (regression parameters and PL CI, but not PL p-value, are available)^28^ and the FC06 macro (regression parameters, PL CI and PL p-value are available)^29^ or in R in the coxphf function^22^. However, irrespective of the statistical methods used, results of studies with less than 400 patients need to be interpreted cautiously because they rarely have sufficient power to detect interaction.

It is important to note that bias depends mainly on the marker effect among patients treated with the standard treatment. Unbiased results are obtained when marker is harmful. However, better performance for a beneficial marker cannot be achieved through recoding of the marker by changing the reference category and estimating $1/\text{HR}_M$ for the standard treatment. This leads to an automatic recoding of the interaction effect to $1/\text{HR}_I$ which just shuffles the different combinations of marker and treatment and changes comparison groups. However, it does not change the number of events in the different subgroups which eventually determines bias. Bias occurs if at least some of the combinations of marker and treatment have few events, caused by a low proportion of patients and/or a low event rate due to a beneficial effect of the marker or a strong treatment effect, or both. Heavy censoring also reduces the number of events and thereby increases bias and the number of non-converged models.

Our simulation study is tightly linked to the situation of markers potentially modifying the effect of systemic BC treatment. We generated and evaluated data which closely resemble actual empirical studies but were limited to qualitative interactions. In additional analyses (data not shown), we evaluated scenarios with quantitative interactions between the marker and the treatment, i.e., with treatment benefits of different magnitude at both marker levels. They showed similar results. This makes our results credible and directly relevant for this specific area. Nevertheless, our results do generally extend to other applications of the Cox proportional hazards model, e.g., cancer at other sites, if one takes the site-specific recurrence rates into account.

The results of our study may partly explain why few predictive markers for BC treatment selection have successfully graduated from preclinical candidate markers to markers used in clinical practice: small sample size and overestimation of standard errors lead to dramatically low power, with the well-known consequences of false-negative results and an increased likelihood of significant results to be false-positive^30^. Appropriate statistical methods can help to remedy the situation somewhat. Applying a Firth corrected Cox model with PL CI instead of a standard Cox model with Wald based CI may help. However, there is still a need for the development of new or the adaptation of standard statistical methods for small studies of predictive biomarkers. Ideally, of course, predictive biomarkers should be investigated in large (enough) studies with sufficient power. To our knowledge, there is currently no software available for calculation of adequate sample size for interaction analyses between two categorical variables based on a Firth-corrected Cox model with PL CIs. However, our script for simulation-based power calculation is available on request from the first author or from our website (http://mhb-fontane-biostatistics.shinyapps.io/Power-CoxFirth/). One can also perform the sample size calculation based on a Cox model with the Power program^31^.

## Additional information

The study by de Boo et al.^10^ was approved by the Ethics Committee of the participating medical institutions in Finland and Sweden and the National Agency for Medicines, Finland. Patients supplied written informed consent to allow the use of their tumour tissue for clinical study related research purposes. The Institutional Review Board at the Helsinki University Hospital, Finland, approved the use of archival tissue for the current translational study.

All involved studies in the study by Knauer et al.^11^ had been approved by the respective institutional review boards.

The study by Kok et al.^12^ was approved by the ethical committees of Lund and Linköping universities. Oral informed consent was registered for all patients.

The study by Schouten et al.^13^ was approved by the Ethical Committee of the University of Heidelberg. Patients enrolled in the trials supplied written informed consent.

The study by Vollebergh et al.^14^ was approved by the Institutional Review Board of the Netherlands Cancer Institute.

All studies were performed in accordance with relevant guidelines and regulations.

### Supplementary Information

Supplementary Information.

## Data Availability

All R scripts are available on request from the first author.
