# Supplementary material for: Cox proportional hazards regression in small studies of predictive biomarkers
Source: Sci Rep. 2024 Jun 20;14:14232. doi: 10.1038/s41598-024-64573-9 (PMC11190253; doi:10.1038/s41598-024-64573-9)
Supplement: Supplementary file 1 — Supplementary Information. [file 41598_2024_64573_MOESM1_ESM.pdf]

Supplementary material to

Cox proportional hazards regression in small studies of predictive biomarkers

Jóźwiak K[1,*], Nguyen VH[1,**], Sollfrank L[1], Linn SC[2,3,4], Hauptmann M[1]

[1]Institute of Biostatistics and Registry Research, Brandenburg Medical School Theodor Fontane, Neuruppin, Germany
[2]Division of Molecular Pathology, The Netherlands Cancer Institute, Amsterdam, The Netherlands
[3]Department of Medical Oncology, The Netherlands Cancer Institute, Amsterdam, The Netherlands
[4]Department of Pathology, University Medical Center, Utrecht, The Netherlands
[*]Corresponding author, Corresponding address: Fehrbelliner Straße 39, 16816 Neuruppin, Germany, katarzyna.jozwiak@mhb-fontane.de
[**] Now at Leibniz Centre for Agricultural Landscape Research (ZALF), Müncheberg, Germany

Figures

Figure S1: Results of the simulation study for a protective ($\text{HR}_M = 0.6$, left panel) and a harmful ($\text{HR}_M = 3$, right panel) marker effect among patients treated with the standard treatment. The treatment HRs were $\text{HR}_{TM_{low}} = 1$ and $\text{HR}_{TM_{high}} = 0.25$, the interaction HR was $\text{HR}_I = 0.25$, the OR between marker and treatment was $\text{OR}_{MT} = 1$, the proportion of patients with high marker level was $p_M = 0.25$, and the proportion of censored patients with low marker level receiving standard treatment was $p_c = 0.5$.

Tables

Table S1. Results of the simulation study for scenarios with extreme bias. The treatment HRs were $\text{HR}_{TM_{low}} = 1$ and $\text{HR}_{TM_{high}} = 0.75$ or $\text{HR}_{TM_{high}} = 0.25$, the interaction HR was $\text{HR}_I = 0.75$ or $\text{HR}_I = 0.25$, the marker effect among patients treated with the standard treatment was $\text{HR}_M = 0.6$, the OR between marker and treatment was $\text{OR}_{MT} = 0.5$ or $\text{OR}_{MT} = 2$, the proportion of patients with high marker level was $p_M = 0.25$, and the proportion of censored patients with low marker level receiving standard treatment was $p_c = 0.2$.

Table S2. Results of the simulation study for no interaction, i.e., $\text{HR}_I = 1$. The treatment HRs were $\text{HR}_{TM_{low}} = 1$ and $\text{HR}_{TM_{high}} = 1$, the marker effect among patients treated with the standard treatment was $\text{HR}_M = 0.6$, the OR between marker and treatment was $\text{OR}_{MT} = 1$, the proportion of patients with high marker level was $p_M = 0.25$, and the proportion of censored patients with low marker level receiving standard treatment was $p_c = 0.2$.

Table S3: Results of the simulation study for a protective ($\mathrm{HR}_M = 0.6$) and a harmful ($\mathrm{HR}_M = 3$) marker effect among patients treated with the standard treatment. The treatment HRs were $\mathrm{HR}_{TM_{low}} = 1$ and $\mathrm{HR}_{TM_{high}} = 0.25$, the interaction HR was $\mathrm{HR}_I = 0.25$, the OR between marker and treatment was $\mathrm{OR}_{MT} = 1$, the proportion of patients with high marker level was $p_M = 0.25$, and the proportion of censored patients with low marker level receiving standard treatment was $p_c = 0.5$.

Table S1: Results of the simulation study for scenarios with extreme bias. The treatment HRs were $\text{HR}_{TM_{low}} = 1$ and $\text{HR}_{TM_{high}} = 0.75$ or $\text{HR}_{TM_{high}} = 0.25$, the interaction HR was $\text{HR}_I = 0.75$ or $\text{HR}_I = 0.25$, the marker effect among patients treated with the standard treatment was $\text{HR}_M = 0.6$, the OR between marker and treatment was $\text{OR}_{MT} = 0.5$ or $\text{OR}_{MT} = 2$, the proportion of patients with high marker level was $p_M = 0.25$, and the proportion of censored patients with low marker level receiving standard treatment was $p_c = 0.2$.

		Bias*						Coverage (%)	Power (%)	
	n	$\widehat{\beta}_{TM_{low}}$	SE($\widehat{\beta}_{TM_{low}}$)	$\widehat{\beta}_{TM_{high}}$	SE($\widehat{\beta}_{TM_{high}}$)	$\widehat{\beta}_I$	SE($\widehat{\beta}_I$)	Wald (PL)	Wald (PL)	N_c
$\text{HR}_{TMhigh} = 0.75$, $\text{HR}_I = 0.75$, $\text{OR}_{MT} = 0.5$										
Cox	200	0	-2.9	0.2	30.2	72.3	22.5	97.7 (97.0)	1.3 (2.4)	7748
	400	0	-1.4	0	7.8	-3.6	7.0	97.2 (96.1)	2.3 (5.0)	9592
	600	0	-0.3	-0.1	-0.7	-20.6	-0.1	96.8 (95.0)	4.5 (8.0)	9914
Firth	200	0	3.2	0.1	20.5	21.4	17.6	98.1 (96.8)	1.0 (3.4)	9879
	400	0	1.0	0	5.5	6.0	5.7	97.4 (95.8)	1.9 (5.0)	9993
	600	0	1.4	0	0.9	-1.0	1.4	96.8 (95.3)	3.7 (6.9)	10000
	n		*M low & T standard*			*M low & T experimental*		*M high & T standard*		*M high & T experimental*
Average number of events	*200*		*12*			*15*		*4*		*2*
	400		*25*			*29*		*7*		*3*
	600		*37*			*44*		*11*		*5*
$\text{HR}_{TMhigh} = 0.25$, $\text{HR}_I = 0.25$, $\text{OR}_{MT} = 2$										
Cox	200	0	-0.7	0.4	61.3	31.2	47.9	98.4 (97.8)	4.9 (7.7)	5315
	400	0	-1.9	0.1	24.9	9.8	22.7	98.0 (97.2)	20.3 (28.0)	8307
	600	0	-0.8	0	12.9	-1.0	12.1	97.8 (96.8)	40.4 (46.9)	9341
Firth	200	0	4.0	0.1	31.6	10.6	28.3	98.9 (97.4)	5.3 (15.6)	9513
	400	0	0.3	0	14.9	2.1	13.8	98.2 (96.6)	21.5 (33.2)	9974
	600	0	0.9	0	7.5	-1.4	7.4	97.8 (96.1)	41.3 (48.6)	9996
	n		*M low & T standard*			*M low & T experimental*		*M high & T standard*		*M high & T experimental*
Average number of events	*200*		*15*			*12*		*2*		*2*
	400		*29*			*25*		*4*		*2*
	600		*44*			*37*		*6*		*3*

*Bias for $\widehat{\beta}_{TM_{low}}$, $\widehat{\beta}_{TM_{high}}$ and relative bias (%) for SE($\widehat{\beta}_{TM_{low}}$), SE($\widehat{\beta}_{TM_{high}}$), $\widehat{\beta}_I$, SE($\widehat{\beta}_I$)

Other parameters were: $\text{HR}_{TM_{low}} = 1$, $\text{HR}_M = 0.6$, $p_M = 0.25$, $p_c = 0.2$

HR, hazard ratio; M, marker; n, number of patients per dataset; N_c, number of converged models; OR, odds ratio; PL, profile likelihood; SE, standard error; T, treatment

Table S2: Results of the simulation study for no interaction, i.e., $\text{HR}_I = 1$. The treatment HRs were $\text{HR}_{TM_{low}} = 1$ and $\text{HR}_{TM_{high}} = 1$, the marker effect among patients treated with the standard treatment was $\text{HR}_M = 0.6$, the OR between marker and treatment was $\text{OR}_{MT} = 1$, the proportion of patients with high marker level was $p_M = 0.25$, and the proportion of censored patients with low marker level receiving standard treatment was $p_c = 0.2$.

		Bias*						Coverage (%)	Type I error (%)	
	n	$\widehat{\beta}_{TM_{low}}$	SE($\widehat{\beta}_{TM_{low}}$)	$\widehat{\beta}_{TM_{high}}$	SE($\widehat{\beta}_{TM_{high}}$)	$\widehat{\beta}_I$	SE($\widehat{\beta}_I$)	Wald (PL)	Wald (PL)	N_c
Cox	200	0	-1.9	0	19.1	0	14.3	98.1 (96.7)	1.9 (3.4)	8859
	400	0	-1.6	0	-0.2	0	0	97.0 (95.3)	3.0 (4.7)	9932
	600	0	-0.5	0	-2.1	0	-1.5	96.1 (95.0)	3.9 (5.0)	9987
Firth	200	0	3.4	0	12.6	0	10.6	98.5 (96.0)	1.5 (4.0)	9910
	400	0	0.7	0	4.2	0	4.1	97.5 (95.9)	2.5 (4.1)	9975
	600	0	1.1	0	1.4	0	1.8	96.6 (95.4)	3.4 (4.6)	9999

*Bias for $\widehat{\beta}_{TM_{low}}$, $\widehat{\beta}_{TM_{high}}$, $\widehat{\beta}_I$ and relative bias (%) for SE($\widehat{\beta}_{TM_{low}}$), SE($\widehat{\beta}_{TM_{high}}$), SE($\widehat{\beta}_I$)

Other parameters: $\text{HR}_{TM_{low}} = 1$, $\text{HR}_{TM_{high}} = 1$, $\text{HR}_I = 1$, $\text{HR}_M = 0.6$, $\text{OR}_{MT} = 1$, $p_M = 0.25$, $p_c = 0.2$

HR, hazard ratio; n, number of patients per dataset; N_c, number of converged models; OR, odds ratio; PL, profile likelihood; SE, standard error

Figure S1: Results of the simulation study for a protective ($\mathrm{HR}_M = 0.6$, left panel) and a harmful ($\mathrm{HR}_M = 3$, right panel) marker effect among patients treated with the standard treatment. The treatment HRs were $\mathrm{HR}_{TM_{low}} = 1$ and $\mathrm{HR}_{TM_{high}} = 0.25$, the interaction HR was $\mathrm{HR}_I = 0.25$, the OR between marker and treatment was $\mathrm{OR}_{MT} = 1$, the proportion of patients with high marker level was $p_M = 0.25$, and the proportion of censored patients with low marker level receiving standard treatment was $p_c = 0.5$.

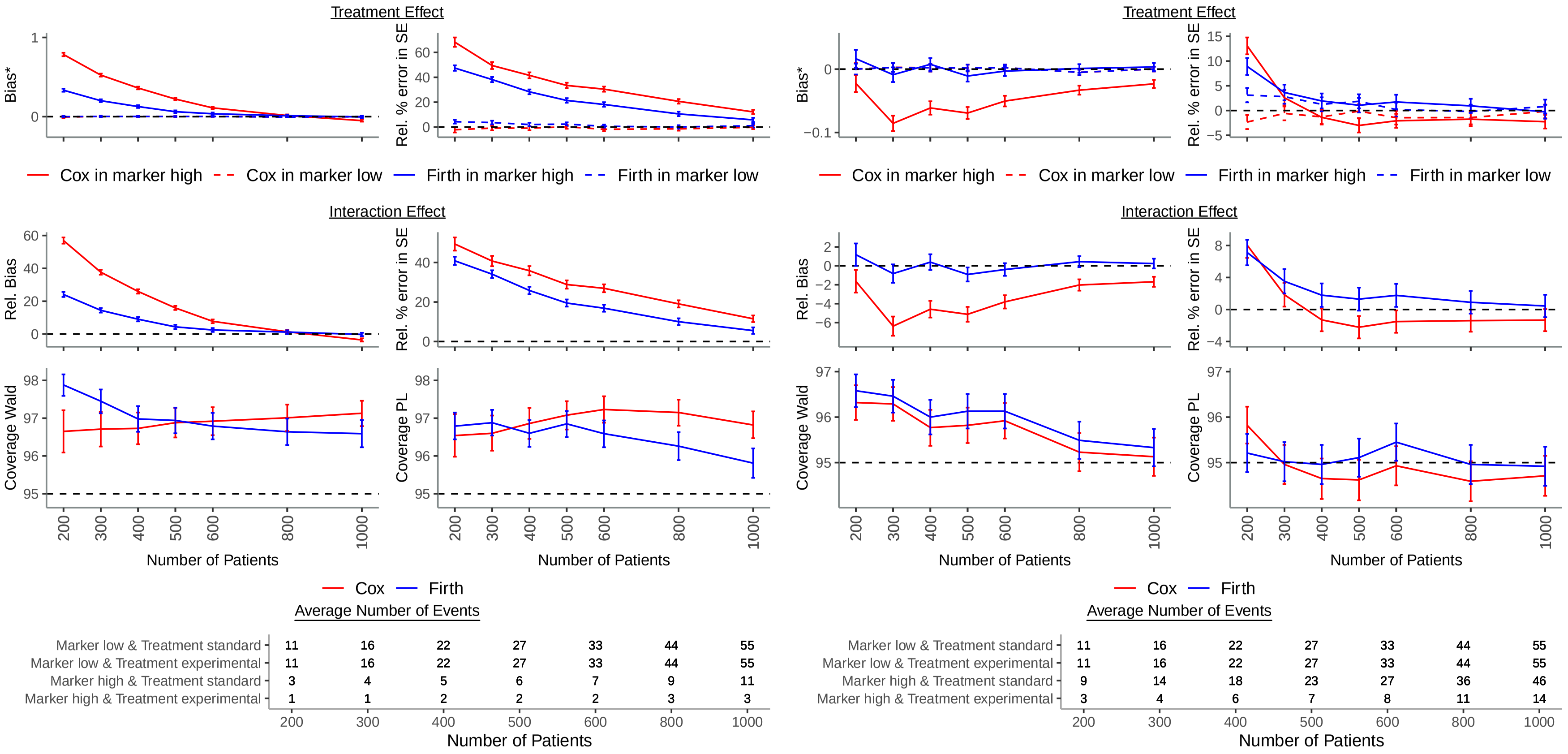

*Curves of bias in marker low group for Cox and Firth model overlap

HR, hazard ratio; OR, odds ratio; PL, profile likelihood; Rel., relative; SE, standard error

Table S3: Results of the simulation study for a protective ($\text{HR}_M = 0.6$) and a harmful ($\text{HR}_M = 3$) marker effect among patients treated with the standard treatment. The treatment HRs were $\text{HR}_{TM_{low}} = 1$ and $\text{HR}_{TM_{high}} = 0.25$, the interaction HR was $\text{HR}_I = 0.25$, the OR between marker and treatment was $\text{OR}_{MT} = 1$, the proportion of patients with high marker level was $p_M = 0.25$, and the proportion of censored patients with low marker level receiving standard treatment was $p_c = 0.5$.

		Bias*						Coverage (%)	Power (%)	
	n	$\widehat{\beta}_{TM_{low}}$	SE($\widehat{\beta}_{TM_{low}}$)	$\widehat{\beta}_{TM_{high}}$	SE($\widehat{\beta}_{TM_{high}}$)	$\widehat{\beta}_I$	SE($\widehat{\beta}_I$)	Wald (PL)	Wald (PL)	N_c
$\text{HR}_M = 0.6$										
Cox	200	0	-2.2	0.8	68.3	56.9	49.3	96.6 (96.5)	1.5 (3.1)	3965
	400	0	-0.9	0.4	41.6	25.9	35.8	96.7 (96.9)	7.1 (14.8)	6884
	600	0	-1.8	0.1	30.5	7.8	26.9	96.9 (97.2)	21.2 (33.9)	8336
Firth	200	0	4.3	0.3	47.4	24.1	40.8	97.9 (96.8)	1.5 (8.3)	9417
	400	0	2.0	0.1	28.3	9.0	25.8	97.0 (96.6)	6.8 (22.4)	9962
	600	0	0.6	0	18.2	2.5	16.9	96.8 (96.6)	20.0 (37.2)	9996
$\text{HR}_M = 3$										
Cox	200	0	-2.3	0	13.1	-1.6	8.0	96.3 (95.8)	34.1 (41.3)	9429
	400	0	-1.3	-0.1	-1.4	-4.6	-1.3	95.8 (94.6)	70.1 (72.7)	9973
	600	0	-1.4	-0.1	-2.1	-3.8	-1.5	95.9 (94.9)	87.8 (88.8)	9999
Firth	200	0	3.1	0	8.9	1.2	7.1	96.6 (95.2)	31.0 (40.6)	9999
	400	0	1.3	0	1.9	0.4	1.8	96.0 (95.0)	67.6 (71.2)	10000
	600	0	0.2	0	1.7	-0.4	1.8	96.1 (95.4)	86.8 (88.1)	10000

*Bias for $\widehat{\beta}_{TM_{low}}$, $\widehat{\beta}_{TM_{high}}$ and relative bias (%) for SE($\widehat{\beta}_{TM_{low}}$), SE($\widehat{\beta}_{TM_{high}}$), $\widehat{\beta}_I$, SE($\widehat{\beta}_I$)
Other parameters: $\text{HR}_{TM_{low}} = 1$, $\text{HR}_{TM_{high}} = 0.25$, $\text{HR}_I = 0.25$, $\text{OR}_{MT} = 1$, $p_M = 0.25$, $p_c = 0.5$
HR, hazard ratio; n, number of patients per dataset; N_c, number of converged models; OR, odds ratio; PL, profile likelihood; SE, standard error
